# Application of a lateral intertubercular sulcus plate in the treatment of proximal humeral fractures: a finite element analysis

**DOI:** 10.1186/s12893-022-01557-4

**Published:** 2022-03-17

**Authors:** Dong Li, WenXue Lv, WenMing Chen, Jing Meng, Song Liu, ZongKang Duan, Bo Yu

**Affiliations:** 1 Shandong University of Traditional Chinese Medicine, Jinan, 250014 China; 2 Department of Orthopedics, Affiliated Hospital of Shandong University of Traditional Chinese Medicine, Jingshi Road 16369, Jinan, 250014 China

**Keywords:** Proximal humeral fracture, Finite element analysis, Lateral intertubercular sulcus plate, Medial support

## Abstract

**Background:**

Inversion deformities caused by insufficient medial support are especially common when the PHILOS locking plate is used to treat proximal humeral fractures. Using finite element analysis, we aimed to compare the biomechanical properties of a PHILOS locking plate (PLP) and a PLP combined with a lateral intertubercular sulcus plate (PLP-LSP) in the fixation of proximal humeral fractures with loss of the medial column.

**Methods:**

After creating a three-dimensional finite element model of a proximal humeral fracture with loss of the medial column, three implant models were established. A full-screw PLP was used in Group A, a PHILOS plate lacking medial screw support and an additional steel plate (MPLP-LSP) were used in Group B, and a full-screw PLP-LSP was used in Group C. The three fixation models were applied to the proximal humeral fracture model, following which horizontal, compressive, and rotational loads were applied to the humerus model. We evaluated structural stiffness and stress distribution in the implant and compared displacement and angle changes among the three models.

**Results:**

Displacement and angle changes were smallest in Group C (PLP-LSP). The implant model used in Group C also exhibited greater structural rigidity, endured less von Mises stress, and was more stable than the models used in Group A and Group B.

**Conclusion:**

An LSP placed at the intertubercular sulcus provides effective lateral and medial support, thereby reducing stress on the PLP and providing better stability with proximal humeral fractures.

## Background

Proximal humeral fractures are frequently encountered in clinical practice, with an incidence of 4–5% [1], which continues to increase each year. Current treatment methods for proximal humeral fractures include intramedullary nailing, internal fixation using a PHILOS locking plate (PLP), and shoulder joint replacement [2]. Although PLPs are commonly used due to their wide scope of application [3], they have been associated with postoperative complications such as poor reduction, varus deformity, screw cutting, nonunion of fractures, infection, and limited function [4]. Barlow et al. [5] followed up 173 patients over 60 years of age with proximal humeral fractures who were treated via internal fixation using a locking plate. They reported failure rates of 26%, 39%, and 45% for two-part (16 cases), three-part (23 cases), and four-part fractures (11 cases), respectively. Varus deformities caused by insufficient medial support are also especially common. Therefore, strengthening the degree of medial support remains an urgent clinical need, as this may help to reduce postoperative complications associated with the use of the PLP [6].

In clinical practice, several methods are used to strengthen medial support, such as allograft fibula implantation [7], titanium mesh implantation [8], bone cement [9], and additional steel plate support plates [10, 11]. Using a 1/3 tubular steel plate as an additional steel plate shaped according to the lateral anatomical structure of the intertubercular sulcus, the authors have developed a method in which the plate is placed on the lateral of the intertubercular sulcus. During the operation, a Kirschner wire is first used for reduction, following which the additional steel plate steel plate is inserted so that it can assist in reduction according to the intertubercular sulcus. At the same time, the Kirschner wire can be removed to facilitate a multi-directional perspective. This surgical method has achieved good therapeutic effects [12], but whether the additional steel plate can strengthen the medial support and enhance stability remains to be verified. Therefore, in the present study, we aimed to evaluate the biomechanical properties and stability of the additional steel plate using finite element analysis.

## Methods

### Establishment of fracture model

**Fig. 1:**
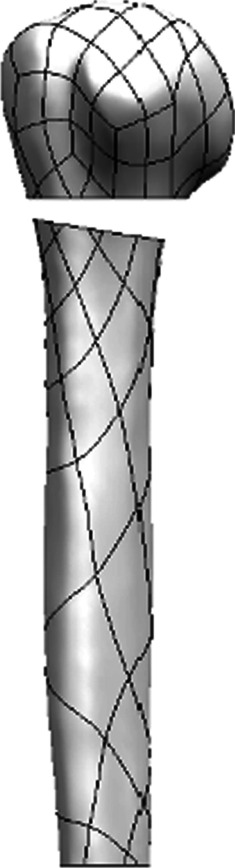
Bone defect area. A bone defect area with a width of 5 mm on the lateral side and 10 mm on the medial side was set at the surgical neck of the humerus to simulate a proximal humeral fracture with an unstable medial column

Standardized computed tomography (CT) data for the humerus were selected to establish a finite element model of the proximal humerus. CT data were obtained from a healthy 27-year-old man. The area of the simulated bone defect at the surgical neck of the humerus extended 5 mm laterally and 10 mm medially. We developed a three-dimensional model of the proximal humeral fracture to simulate instability of the medial column (Fig. 1). The distinction between the cortical and cancellous bone was based on the CT gray value, and the ranges of gray values for the cortical and cancellous bone were 662–1841 HU and 148–661 HU, respectively. The types of implants included the PLP and lateral additional steel plate of the intertubercular sulcus. The PLP was 90 mm long and 3 mm thick, with a screw length of 3.5 mm. The additional steel plate was 50 mm long and 2.5 mm thick, with a screw length of 2.5 mm. The arc of the plate was designed according to the anatomical structure of the lateral of the intertubercular sulcus.

### Implant assembly

**Fig. 2:**
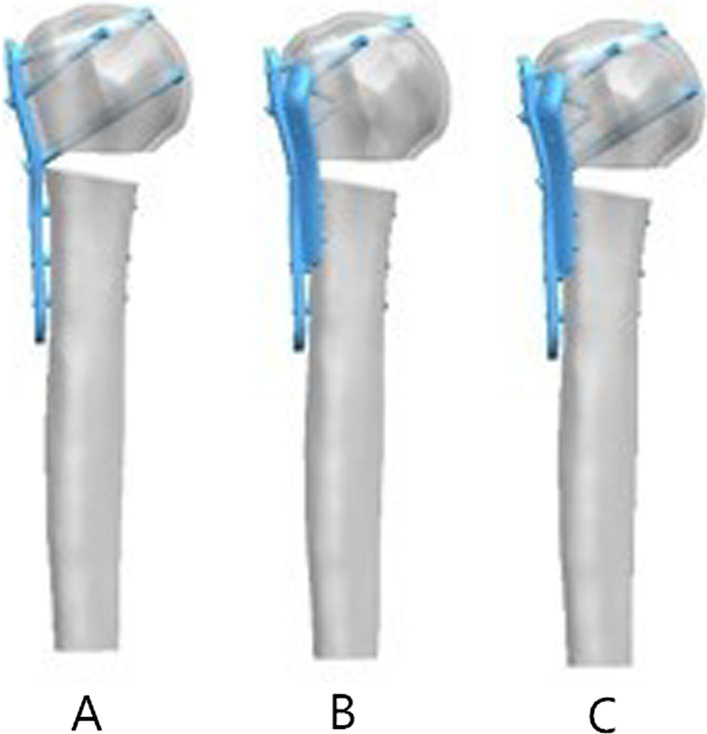
Three implant models. **A** PHILOS plate (PLP). **B** PHILOS plate lacking medial screw support and lateral intertubercular sulcus plate (MPLP-LSP). **C** PHILOS plate and lateral intertubercular sulcus plate (PLP-LSP)

The PLP was assembled on the fracture model in accordance with the standard method, and the upper end was placed 5 mm from the apex of the major tubercle. The additional steel plate was tightly attached to the lateral of the intertubercular sulcus, and the upper end was placed 8 mm from the apex of the major tubercle. In group A all proximal rows (A–D)were occupied, Group B only the four screws in row A and B were inserted while inserting the additional steel plate. In group C all proximal rows (A–D)were occupied while inserting the additional steel plate. Two screws each were inserted at the proximal and distal ends of the additional steel plate.(Fig. 2). The threads of the locking screws were omitted to simplify the models. The PLP model included 9566 elements and 16,117 nodes. The additional steel plate model included 1781 elements and 3314 nodes.

### Setting of parameters

**Fig. 3:**
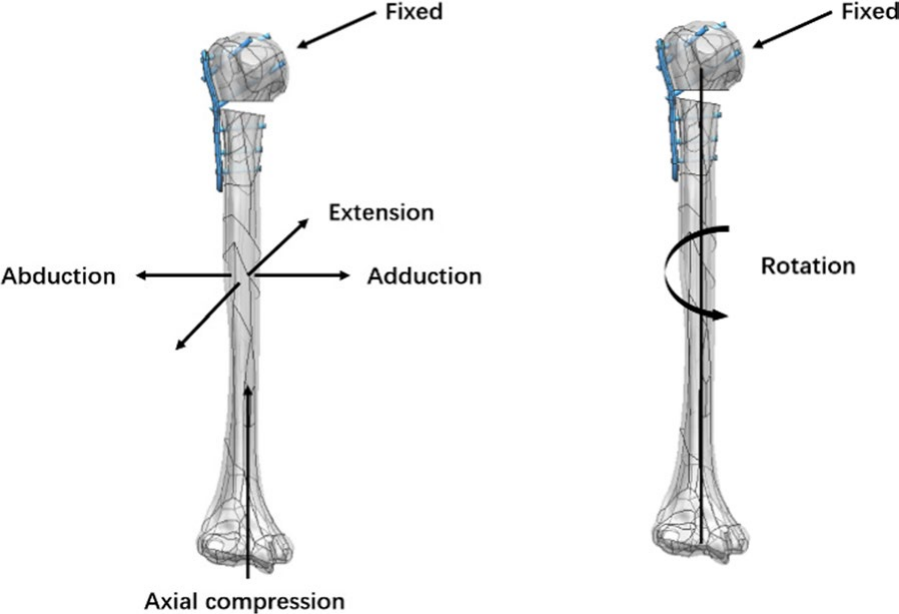
Load application. Compressive and rotational loads were applied to the humerus model to simulate the functions of the shoulder joint, including abduction, adduction, flexion, extension, axial compression, and internal and external rotation

Finite element analysis was performed using Abaqus 6.14 software (3DS, Waltham, MA). Linear elastic isotropic material properties were assigned to all models and materials placed. The elastic modulus of the normal cortical bone was set to 8844 MPa, that of cancellous bone was set to 660 MPa, and that of the built-in steel plate was set to 114,000 MPa. The interface between the humeral head and glenoid was fixed in all models of the proximal humeral fracture. The contact behavior of the plate/locking-screw and bone/locking-screw interfaces was defined as fully fixed. The contact behavior of the plate/bone and cortical-screw/bone interfaces was defined as surface-to-surface. All contact elements were defined as deformable elements. The analyses were performed by assuming frictionless interactions to simplify the contact phenomena. Compression and rotation loads were applied to the humerus model to simulate the functions of the shoulder joint, including abduction, adduction, flexion, extension, axial compression, and internal and external rotation (Fig. 3). Loads of 100 N were applied to the four directions of the humeral shaft to simulate the effects of shoulder muscle abduction, adduction, flexion, and extension, and a load of 200 N was applied to the end of the humerus to simulate axial compression. A torque of 7.5 Nm was applied to the end of the humerus to simulate internal and external rotation [13].

### Evaluation indices

#### Fracture stability

**Fig. 4:**
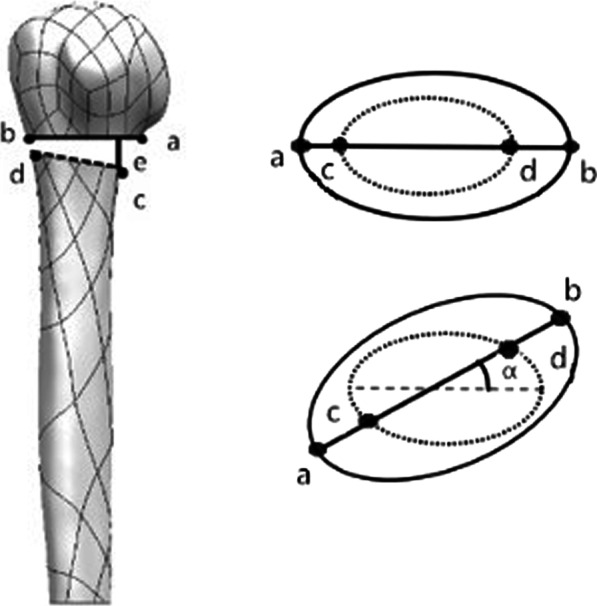
Stability. The stability of the fracture region under horizontal and compressive loads was assessed based on the distance covered by the medial fracture gap (line *e*). The angular variation between the proximal and distal fracture gap was determined to assess regional rotational stability (angle α)

The vertical distance between points *c* and *ab* is defined as *e*, where *c*, *a,* and *b* are the distal medial, proximal medial, and lateral points, respectively, of the fractured end, and *e* is the displacement of the gap between the fracture ends. The stability of the fractured end was evaluated by measuring the change in the displacement (*e*) of the gap between the fracture ends (Fig. 4).

#### Rotational stability

The rotational stability of the humeral head was evaluated by measuring the change in the angle (α: the angle of the two straight lines; *ab* and *cd*) between the proximal and distal fractures of the fractured end [14] (Fig. 4).

#### Stress

For each model, we measured the equivalent pressure distribution (von Mises stress) and maximum stress on the steel plate to evaluate the degree of stress.

## Results

### Construct stiffness

The compression and rotation stiffness values of the three models were calculated using finite element analysis (Table 1). Compression and rotation stiffness values were 39.84 N/mm and 110.20 Nm/Rad in Group A (PLP), 43.67 N/mm and 153.50 Nm/Rad in Group B (MPLP-LSP), and 66.67 N/mm and 204.67 Nm/Rad in Group C (PLP-LSP), respectively.

**Table 1:** Construct stiffness

Group	Compression stiffness (N/mm)	Rotational stiffness (Nm/Rad)
A	39.84	110.20
B	43.67	153.50
C	66.67	204.67

Group A (PLP): Full-screw PHILOS plate; Group B (MPLP-LSP): PHILOS plate without medial screw support plus additional steel plate; Group C (PLP-LSP): full-screw PHILOS plate plus additional steel plate.

### Implant stress

**Fig. 5:**
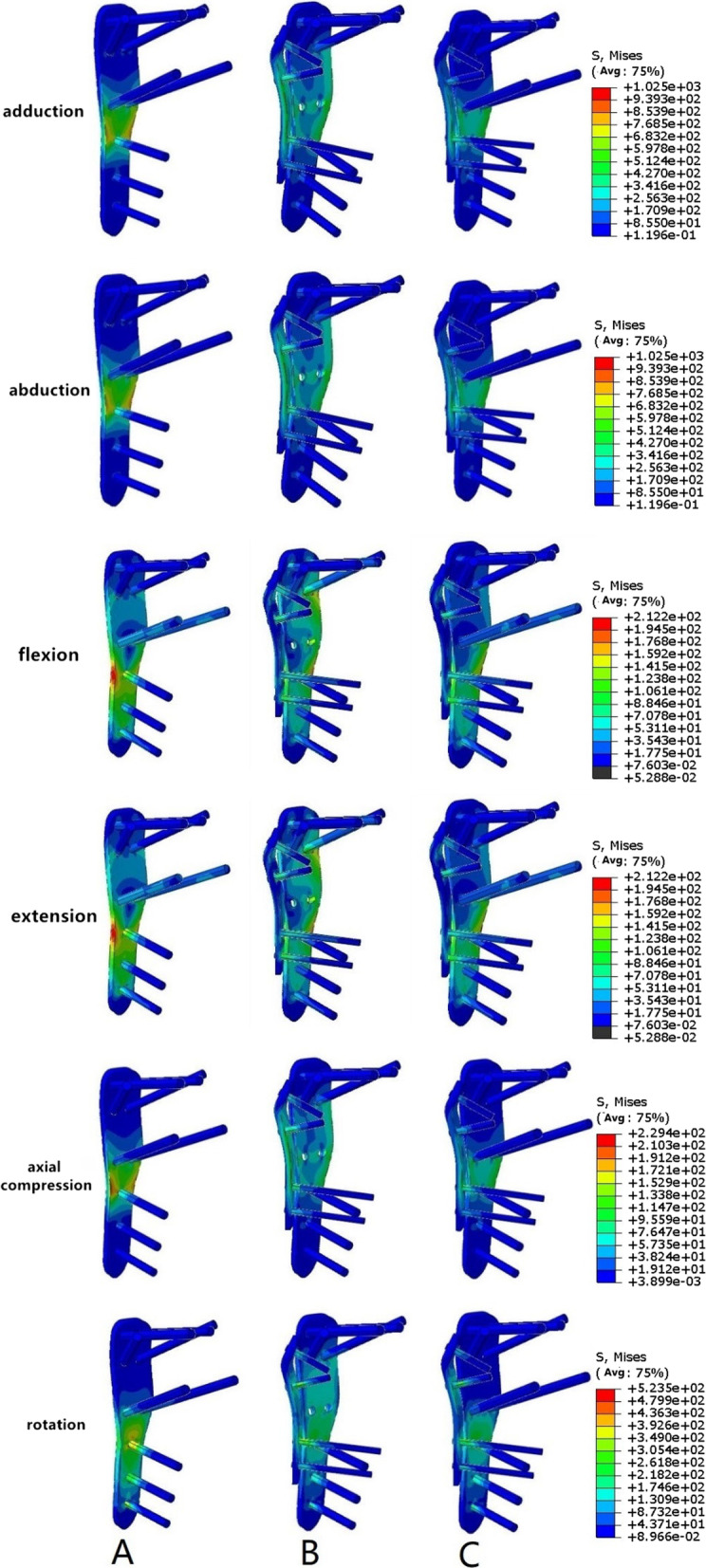
The maximum von Misses stress and stress distribution. Group A (PLP): Full-screw PHILOS plate; Group B (MPLP-LSP): PHILOS plate without medial screw support plus additional steel plate; Group C (PLP-LSP): full-screw PHILOS plate plus additional steel plate

The maximum equivalent stress, stress distribution, and maximum von Mises stress under different load conditions were calculated for each model using finite element analysis (Table 2, Fig. 5).

**Table 2:** Maximum von Mises stress (MPa)

Group	Adduction	Abduction	Flexion	Extension	Axial compression
A	1025	1025	212.2	212.2	229.4
B	892.6	892.6	283.8	283.8	198.6
C	858.6	858.6	204.8	204.8	164.1

In Group A, stress was concentrated near the support screw area during shoulder joint movement. When compared with those in other groups, the stress values under different load conditions were lowest in Group C, suggesting that the additional steel plate greatly dispersed the stress, thereby reducing maximum stress.

Group A (PLP): Full-screw PHILOS plate; Group B (MPLP-LSP): PHILOS plate without medial screw support plus additional steel plate; Group C (PLP-LSP): full-screw PHILOS plate plus additional steel plate.

### Displacement changes

**Fig. 6:**
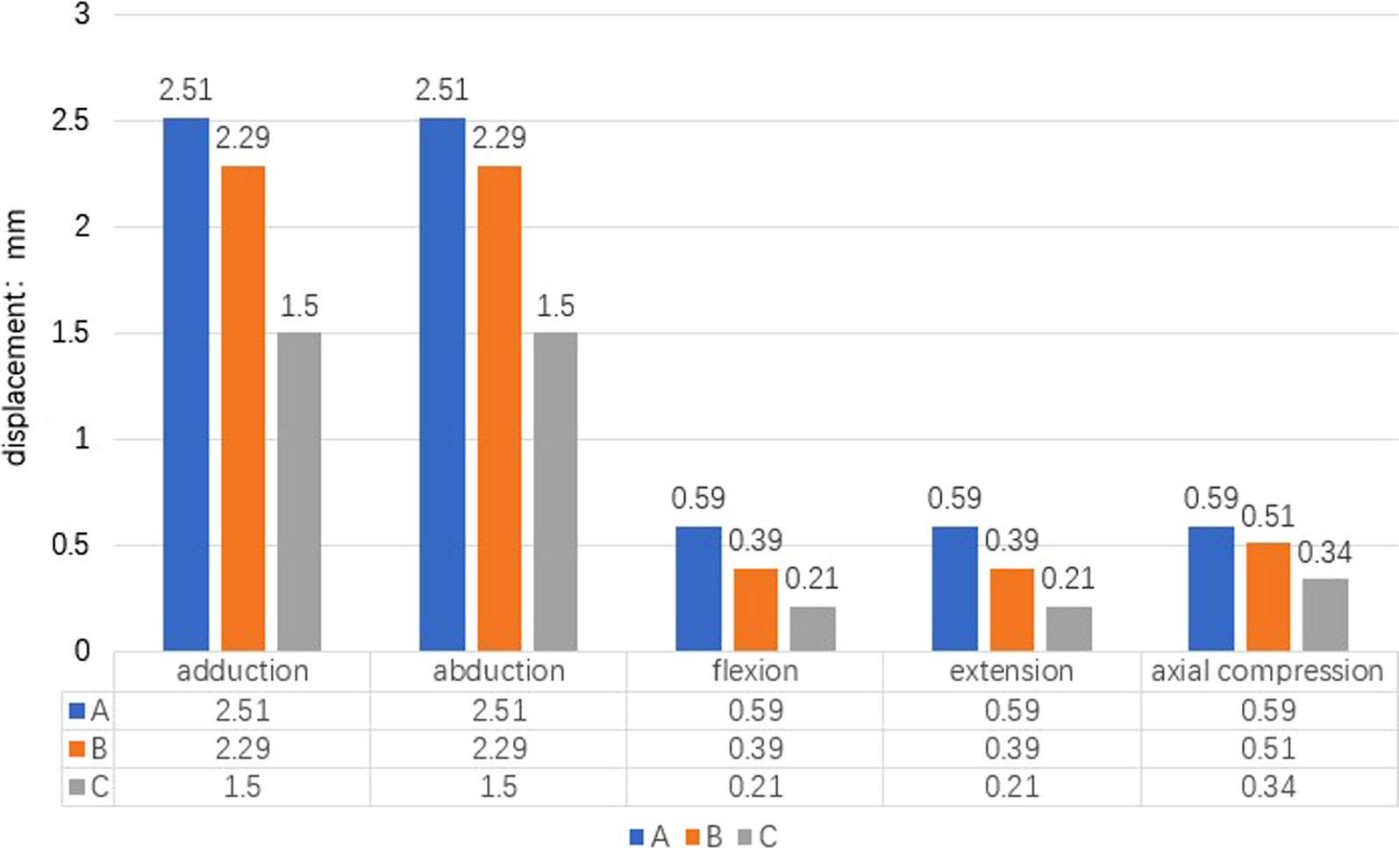
Changes in the displacement of the fracture region under different loading conditions. Group A (PLP): Full-screw PHILOS plate; Group B (MPLP-LSP): PHILOS plate without medial screw support plus additional steel plate; Group C (PLP-LSP): full-screw PHILOS plate plus additional steel plate

The displacement observed during different simulated activities for each model is displayed in Fig. 6.

### Angle changes

The angle changes measured during rotation were 3.9° in Group A (PLP), 2.8° in Group B, and 2.1° in Group C (Table 3).

**Table 3:** Angle changes during rotation

Group	A	B	C
Angle changes	3.9°	2.8°	2.1°

## Discussion

In the present study, we used finite element analysis to explore the biomechanical properties of a lateral plate at the intertubercular groove in a model of proximal humeral fracture with loss of the medial column. Our findings indicated that greater structural stiffness under axial compression and rotational load was associated with increased ability of the internal fixation system to prevent varus displacement of the humeral head. Our comparison between Groups A and B indicated that the additional steel plate can completely replace the support function of the supporting screws while ensuring greater structural rigidity. Results from Group C further suggest that combining the additional steel plate with the original PLP leads to even greater structural rigidity and stability than that observed in Groups A and B. Regardless of the force applied in the horizontal, vertical, and torsional directions, the changes in displacement and angle in Group C were only one half of those observed in Group A, indicating that the steel plate significantly increases the stability and medial support of the original PHILOS system.

The maximum von Mises stress on the internal fixation reflects the route by which load transfer occurs with different internal fixation methods, and greater von Mises stress is indicative of greater torsional force. Thus, after a long period of repeated twisting, the internal fixation is the part most likely to fail. The maximum von Mises stress values were smallest in Group C under various loads when compared with those in the other groups. Therefore, these results indicate that the additional steel plate can provide a better internal support, effectively disperse stress, reduce the risk of internal fixation failure, and enhance the stability of the internal fixation.

Screw cutting and varus displacement of the humeral head are the most common surgical complications of open reduction and internal fixation in patients with proximal humeral fractures. Lack of a medial support has been cited as an important reason for postoperative complications and surgical failure [15], and the two medial support screws of the PLP are particularly important for ensuring medial support of the proximal humerus [16, 17]. A comparative study by Shen et al. [18] reported that placement of the medial support screw greatly reduced screw cutting, varus deformity, and the probability of a secondary surgery. In clinical practice, various methods such as autologous or allogeneic fibula implantation, titanium mesh implantation, and the use of an additional steel plate are used to compensate for the effect of the medial support screw and strengthen medial support [7–11]. The selection, treatment, and placement of fibula grafts and titanium mesh require that orthopedic doctors have high technical experience. In addition to their high cost and the increased risk of infection and disease transmission, the availability of the materials required for these methods is limited [19]. Although an additional steel plate placed on the inner side of the proximal humerus can directly provide effective medial support, the medial approach is not easy to learn due to the complex anatomy of the neurovascular structure. As improper techniques can easily lead to iatrogenic nerve and blood vessel damage, the medial plate approach for proximal humeral fractures has not been clinically promoted. With our technique, the additional steel plate is pre-bent (1/3 tubular steel plate) according to the anatomical shape of the lateral of the intertubercular sulcus. This is because the intertubercular sulcus can be used as a landmark to assist in anatomical reduction [20], and the lateral side of the intertubercular sulcus can be easily exposed without additional trauma during the operation. Use of the conventional anteromedial approach can reduce the risk of damaging muscle nerve branches [21]. After the additional steel plate is placed for temporary fixation during the operation, the Kirschner wire used to maintain the reduction can be removed, which is convenient for multi-angle fluoroscopy and shortens the operation time.

Since Brekelmans et al. first introduced the finite element method in biomechanics research [22], the application of finite element analysis in orthopedic biomechanics has evolved, and it is widely used to evaluate new implants or materials, strain and stress distribution, and load transfer between objects and bones [23]. However, given the complex structure of the shoulder joint, it is impossible to accurately simulate the real boundary conditions of the interactions of all muscles and ligaments. Our research aims to simplify the study of the shoulder joint by ignoring the interactions of muscles, ligaments, bones, and other surrounding structures [24]. However, although finite element analysis can simulate the properties of various bone materials and load forces in various directions, it does not fully reflect the real-world situation due to differences in bone density and fracture types among patients.

## Conclusion

Our findings demonstrate that an LSP placed at the intertubercular sulcus provides effective lateral and medial support, thereby reducing stress on the PLP and providing better stability in a finite element model. The use of the PLP-LSP method may represent a novel strategy for the treatment of proximal humeral fractures.

## Data Availability

The datasets used and/or analyzed during the current study are available from the corresponding author on reasonable request.

## Abbreviations

CT: Computed tomography
Full-screw: The proximal 6 screws are fully assembled
PLP: Full-screw PHILOS locking plate
MPLP-LSP: PHILOS plate lacking medial screw support plus additional steel plate
PLP-LSP: Full-screw PHILOS locking plate plus additional steel plate
